# Biological, social, and healthcare factors for death due to influenza A(H1N1) during the 2009 epidemic in Brazil

**DOI:** 10.11606/s1518-8787.2024058005855

**Published:** 2024-07-22

**Authors:** Jose Ueleres Braga, Ana Freitas Ribeiro

**Affiliations:** I Universidade do Estado do Rio de Janeiro Instituto de Medicina Social Hesio Cordeiro Departamento de Epidemiologia Rio de Janeiro RJ Brasil Universidade do Estado do Rio de Janeiro. Instituto de Medicina Social Hesio Cordeiro. Departamento de Epidemiologia. Rio de Janeiro, RJ, Brasil; II Fundação Oswaldo Cruz Escola Nacional de Saúde Pública Sergio Arouca Departamento de Epidemiologia de Doenças Infecciosas Rio de Janeiro RJ Brasil Fundação Oswaldo Cruz. Escola Nacional de Saúde Pública Sergio Arouca. Departamento de Epidemiologia de Doenças Infecciosas. Rio de Janeiro, RJ, Brasil; III Secretaria de Estado da Saúde de São Paulo Instituto de Infectologia Emílio Ribas São Paulo SP Brasil Secretaria de Estado da Saúde de São Paulo. Instituto de Infectologia Emílio Ribas. São Paulo, SP, Brasil; IV Universidade Municipal de São Caetano do Sul Faculdade de Medicina São Paulo SP Brasil Universidade Municipal de São Caetano do Sul. Faculdade de Medicina. São Paulo, SP, Brasil

**Keywords:** Influenza A Virus, H1n1 Subtype, Disease Outbreaks, Risk Factors, Case-Control Studies

## Abstract

**OBJECTIVE:**

To identify risk factors for death from influenza A(H1N1), including the effectiveness of the vaccine against influenza A(H1N1) concerning mortality.

**METHODS:**

A case-control of incident cases of influenza A(H1N1) reported in the epidemiological information systems of the states of São Paulo, Paraná, Pará, Amazonas, and Rio Grande do Sul was conducted.

**RESULTS:**

305 participants were included, 70 of them cases and 235 controls, distributed as follows: Amazonas, 9 cases/10 controls; Pará, 22 cases/77 controls, São Paulo, 19 cases/49 controls; Paraná, 10 cases/54 controls; Rio Grande do Sul, 10 cases/45 controls. These participants had a mean age of 30 years, with 33 years among cases and 25 years among controls. There was a predominance of females both among the cases and controls. Biological (age), pre-existing diseases (congestive heart failure, respiratory disease, and diabetes mellitus), and care factors (ICU admission) associated with death from influenza A(H1N1) were identified.

**CONCLUSION:**

The risk factors identified in this investigation not only allowed subsidizing the elaboration of clinical conducts but also indicate important aspects for facing “new” influenza epidemics that are likely to occur in our country.

## INTRODUCTION

In April 2009, the CDC reported two cases of febrile respiratory illness in children in Southern California with laboratory diagnosis of infection with a virus genetically similar to swine influenza A(H1N1). The virus contains a gene segment not previously identified in human and swine influenza. The two children had no contact with the pigs. Another six cases have been reported to the California and Texas departments of public health. At the same time, samples from patients in Mexico confirmed the same viral subtype^1^.

On April 12, 2009, Mexico recorded an outbreak of acute respiratory disease in Gloria, Veracruz, characterized by a high attack rate (28.5%). In March and April, 47 cases of severe pneumonia with 12 deaths were identified in Mexico City, San Luis de Potosi, and other cities. In four samples, the new viral subtype of influenza A(H1N1) was identified^2^.

On June 11, 2009, the World Health Organization (WHO) raised the global alert level for the pandemic caused by the new influenza A(H1N1) virus *—* swine lineage — to Phase 6 (Pandemic). The action occurred because of the rapid spread of the virus and not because of the severity of the disease^1^. The vast majority of cases evolved to cure. However, it was expected that the number of cases, hospitalizations, and deaths would increase at the end of the epidemic^3^. In August 2010, the WHO declared the end of the pandemic, with approximately 18,500 deaths, moving into the post-pandemic phase, with seasonal transmission of the virus^4^.

Sustained transmission in Brazil was established on July 16, 2009. From that date, only cases of Severe Acute Respiratory Illness (SARI) were reported, hospitalized, and treated with oseltamivir. Outpatient cases, if considered patients at risk (comorbidities, pregnant women, immunosuppressed), could also receive specific treatment up to 48 hours after the onset of symptoms, after medical evaluation. However, in this case, there was no clinical sample collection for diagnosis and no record in the notifiable health information system (SINAN)^5^.

At that time, it was believed to be necessary to maintain monitoring of possible changes in the profile of viral circulation and the worsening of the clinical picture of cases of influenza A(H1N1) because of the concomitant circulation of different subtypes of influenza viruses and new evidence of predictive factors for severity and deaths related to this new disease^6^.

Therefore, conducting studies that evaluated possible risk factors for death from Influenza A(H1N1) and for death in pregnant women was essential for a better understanding of this epidemic. This study aimed to identify risk factors for death from influenza A(H1N1), a new viral subtype associated with Severe Acute Respiratory Illness (SARI), including the effectiveness of the H1N1 vaccine in terms of mortality, based on clinical epidemiological data of cases reported to the Brazilian epidemiological surveillance system.

## METHODS

This case-control study included incident cases of influenza A(H1N1) reported in the epidemiological information systems of the states of São Paulo, Paraná, Pará, Amazonas, and Rio Grande do Sul. The study population included children, adolescents, and adults with symptoms compatible with suspected infection by the influenza A(H1N1) virus treated at the unified health system.

Individuals included as cases had confirmed influenza A(H1N1) infection with fever, respiratory symptoms, and a positive confirmatory test for the presence of the virus and death was registered in the Epidemiological Surveillance System for Influenza (SIVEP-Flupe) or the Information System on Mortality (SIM). These individuals were evaluated in loco, i.e., their medical records were evaluated.

A time-paired case-control design was used^7^ by the epidemiological week of hospitalization, and H1N1 notification records were drawn from the SIVEP-Flu database according to the ratio of four controls for each case^8^.

First, the medical records were evaluated, and the questionnaire for the first two selected controls was completed. The interviewer sent the completed questionnaires to the field supervisor, who also evaluated them to verify that the controls fulfilled the case definition. If they did not, the other randomly selected controls had their medical records evaluated. Two controls were sought per case, and a maximum of four medical records of possible patients eligible for control were evaluated.

Therefore, the sources of data were 1) medical records, 2) data or information obtained from family members through home visits, and 3) data from SIVEP-Flu. A standardized form was used to collect data from medical records and home interviews, both for cases and controls.

The variables studied were related to 1) time: date of symptom onset, date of first consultation, date of hospitalization, and date of death; 2) place: place of residence, place of outpatient and hospital care; 3) person: age, sex, education, self-reported race/color, occupation, underlying diseases, and vaccination; 4) clinical and laboratory characteristics: initial clinical and laboratory evaluation, radiological imaging, use of O2, presence of comorbidities, pregnancy (when applicable), associated infections, other diagnoses during hospitalization, complications during hospitalization, treatment with oseltamivir (dose, time of use and interval of use), cause of death, data from the death certificate, necropsy report (when available), anatomopathological reports, and relevant laboratory results (cultures performed).

The size was calculated on the basis of a 5% alpha error and 20% beta error and indicated the need for 80 A(H1N1) influenza deaths and 320 controls, but if the association estimate had a magnitude greater than 3.0, fewer participants would be needed.

This study was approved by the Research Ethics Committee of the Institute of Social Medicine of the State University of Rio de Janeiro on December 4, 2009 (CAEE 0030.1.259.000-09).

## RESULTS

A total of 305 participants were included, of those 70 were cases and 235 were controls. Two federal units of the original project were replaced (Rio de Janeiro and Minas Gerais) by the states of Amazonas and Pará. The number of cases and controls in each federal unit was as follows: Amazonas, 9 cases/10 controls; Pará, 22 cases/77 controls, São Paulo, 19 cases/49 controls; Paraná, 10 cases/54 controls; and Rio Grande do Sul, 10 cases/45 controls.

Cases and controls were recruited in these federated units in 2010 and 2011, and most participants experienced symptom onset in the first year of the study. At that time, the vaccine had already been applied to the population, and this would also allow the evaluation of the effect of this intervention on the risk of death from influenza A(H1N1).

The states of Amazonas and Pará included the study participants later because the number of deaths in the country had declined in such a way that it negatively influenced recruitment in these states.

These participants had a mean age of 30 years, with 33 years among cases and 25 years among controls. There was a predominance of females both among the cases and controls. The age group with the highest frequency of participants was 18 to 49 years old (Table 1).

**Table 1:** Distribution of demographic characteristics and risk conditions of participants in the case-control study for risk factors for death due to influenza A(H1N1).

Variables	Cases		Controls		Total	p-value
	
	n	%	n	%		
Sex						
Male	18	18.0	82	82.0	100	0.1509
Female	52	25.4	153	74.6	205	
Age (years)						
≤ 18	15	14.9	86	85.1	101	0.0106
19–49	36	25.9	103	74.1	139	
≥ 50	16	37.2	27	62.8	43	
Mean age	33.6		25.4			0.0009
Median age	34		23			
Risk conditions						
Pregnant	14	26.9	38	73.1	52	0.0711
Quarter						
1	0	0.0	5	100.0	5	
2	3	15.0	17	85.0	20	
3	10	38.5	16	61.5	26	
Puerperium	1	25.0	3	75.0	4	0.0002
Obesity	10	55.6	8	44.4	18	0.1036
Cardiovascular disease	7	43.8	9	56.3	16	0.0010
Hypertension	15	50.0	15	50.0	30	0.1835
Coronary Disease	1	100.0	0	0.0	1	0.1835
Cerebrovascular disease	2	100.0	0	0.0	2	0.0279
Heart Failure	4	80.0	1	20.0	5	0.0084
Diabetes	7	87.5	1	12.5	8	0.0001
Thyroid disease	3	37.5	5	62.5	8	0.6074
Respiratory disease	17	38.6	27	61.4	44	0.0250
Asthma	6	21.4	22	78.6	28	0.9703
COPD	6	46.2	7	53.8	13	0.1011
Tuberculosis	1	50.0	1	50.0	2	0.6119
Cystic fibrosis	0		0		0	
Liver disease	2	40.0	3	60.0	5	0.6488
Hemoglobinopathy	0		0		0	
DAI	1	25.0	3	75.0	4	0.7615
Lupus	0	0.0	1	100.0	1	0.6123
Rheumatoid arthritis	0	0.0	2	100.0	2	0.5519
Others DAI	1	50.0	1	50.0	2	0.4921
Kidney disease	1	100.0	0	0.0	1	0.1341
Others diseases	9	21.4	33	78.6	42	0.9645
HIV	1	16.7	5	83.3	6	0.9049
Organ transplantation	0	0.0	1	100.0	1	0.8220
Neoplasm	1	25.0	3	75.0	4	0.9767
Immunosuppressive drug	1	16.7	5	83.3	6	0.7986

COPD: chronic obstructive pulmonary disease; DAI: autoimmune disease; HIV: human immunodeficiency virus.

Regarding the clinical risk factors for death from influenza A(H1N1) between cases and controls, differences in the study for the occurrence of some clinical conditions were also observed in the scientific literature. Cases most often had the following clinical conditions: obesity, systemic arterial hypertension, cerebrovascular disease, congestive heart failure, diabetes mellitus, and respiratory disease. Pregnancy was more frequent among cases than controls, but this difference was not statistically significant at the 5% level (Table 1).

The most frequent signs and symptoms were fever (94.1%), cough (91.1%), dyspnea (80.3%), and headache (33.8%). Regarding the distribution between cases and controls, there were no important differences regarding the characteristic symptoms of respiratory disease. However, dyspnea was more frequent in those who died (Table 2).

**Table 2:** Distribution of signs and symptoms. hospitalization and procedures of participants in the case-control study for risk factors for death due to influenza A(H1N1).

Variables	Cases		Controles		Total	p-value
	
	n	%	n	%		
Signs and symptoms						
Fever	62	21.6	225	78.4	287	0.0053
Cough	62	22.3	216	77.7	278	0.2067
Dyspnea	67	27.3	178	72.7	245	0.0004
Headache	10	9.7	93	90.3	103	0.0001
Chill	9	26.5	25	73.5	34	0.2774
Sore throat	10	13.9	62	86.1	72	0.1776
Arthralgia	2	7.1	26	92.9	28	0.0658
Myalgia	16	17.0	78	83.0	94	0.1602
Conjunctivitis	0	0.0	7	100.0	7	0.1760
Runny nose	13	13.8	81	86.2	94	0.0127
Diarrhea	13	34.2	25	65.8	38	0.0611
Vomiting	11	19.0	47	81.0	58	0.3556
Seizures	1	50.0	1	50.0	2	0.2407
Asthenia	10	14.9	57	85.1	67	0.1366
Lack of appetite	7	16.3	36	83.7	43	0.1288
Irritability	1	25.0	3	75.0	4	0.5550
Others	39	24.1	123	75.9	162	0.7930
Hospitalization						
Transfer from another service	36	41.4	51	58.6	87	0.0000
ICU admission	60	64.5	33	35.5	93	0.0000
Use of Oseltamivir	65	23.8	208	76.2	273	0.4240
Complications	59	67.8	28	32.2	87	0.0000
Shock	42	89.4	5	10.6	47	0.0000
Sepsis	45	80.4	11	19.6	56	0.0000
Clotting Disease	8	88.9	1	11.1	9	0.0000
Acute Respiratory Distress Syndrome	36	80.0	9	20.0	45	0.0000
Pleural disease	10	66.7	5	33.3	15	0.0001
Pulmonary hemorrhage	10	90.9	1	9.1	11	0.0000
Other lung diseases	23	59.0	16	41.0	39	0.0000
Heart failure	14	93.3	1	6.7	15	0.0000
Kidney disease	25	86.2	4	13.8	29	0.0000
Procedures						
Use of antibiotics	66	25.9	189	74.1	255	0.0137
Oxygen therapy	58	36.5	101	63.5	159	0.0000
Non-invasive ventilation	29	64.4	16	35.6	45	0.0000
Invasive ventilation	53	81.5	12	18.5	65	0.0000
24-hour ventilation requirement	47	79.7	12	20.3	59	0.0000
Respiratory physiotherapy	45	36.6	78	63.4	123	0.0000
Anticoagulant	29	58.0	21	42.0	50	0.0000
Nasogastric tube	57	81.4	13	18.6	70	0.0000
Central Venous Catheter	41	80.4	10	19.6	51	0.0000
Tracheostomy	17	70.8	7	29.2	24	0.0000

ICU: intensive care unit.

The treatment that the cases and controls underwent was quite different than expected. There was no difference concerning the use of oseltamivir because this medication was available and used very frequently at that time. However, ICU admission and transfer to another service occurred more among cases compared with controls. The most observed clinical complications were shock and sepsis, in practically two-thirds of the patients who died. All other complications were observed among the cases (Table 2).

The therapeutic procedures used in cases and controls were also different, being more frequent in individuals who died. The use of antibiotics was different in the two groups, indicating that secondary infections may have occurred more frequently between cases. We highlight the use of invasive and noninvasive ventilation, anticoagulants, and nasogastric tubes. These findings indicate that patients who died received intensive treatment and that these procedures were available to individuals in the control group (Table 2).

The vaccination status of the participants differed between the study sites. Participants from Paraná were more vaccinated (15.6%) than those from the other States: Amazonas (5.3%), Pará (1%), and Rio Grande do Sul (6.3%). In the state of São Paulo, no participant had received the vaccine. On average, only 5% of the study participants were vaccinated.

Regarding pre-existing diseases, it can be said that they influence the prognosis of patients, increasing the risk of death from influenza A(H1N1). It is noticed that not only chronic respiratory diseases but also systemic diseases are associated with death, such as diabetes mellitus and congestive heart failure. The analysis of the joint influence of these morbid conditions revealed that, although respiratory conditions are important, they are not the greatest risk factors (Table 3). Our study did not detect that morbid conditions characterized by immunosuppression had an increased risk for our outcome.

**Table 3:** Vaccination status and biological risk factors for death due to influenza A(H1N1).

Risk factor	Association measures			

	OR_crude_	95%CI	OR_adjusted_	95%CI
Biological characteristics				
Female sex	1.417	0.794–2.528	1.360	0.718–2.591
Age (years)	1.026	1.011–1.041	1.020	1.007–1.038
Are you obese?	4.091	1.594–10.502	3.246	1.221–8.632
Vaccination status				
Did you vaccinate for Influenza?	1.106	0.345–3.540	0.829	0.222–3.097
Pre-existing diseases				
Cardiovascular diseases	3.248	1.266–8.333	0.682	0.165–2.823
Systemic arterial hypertension	3.636	1.698–7.786	2.051	0.769–5.470
Cerebrovascular disease	6.760	0.604–75.650	1.191	0.056–25.050
Congestive heart failure	21.492	2.543–181.625	14.425	1.032–201.538
Diabetes mellitus	29.538	3.628–240.454	10.768	1.075–107.819
Respiratory tract diseases	2.301	1.195–4.432	1.880	0.855–4.134
COPD	3.545	1.200–10.468	1.701	0.454–6.377
Hospital care				
Has it been transferred from the service?	3.645	2.101–6.324	2.264	1.099–4.664
Were you admitted to the ICU?	37.080	17.387–79.074	32.742	15.250–70.300

ICU: intensive care unit; COPD: chronic obstructive pulmonary disease.

To answer the research question of “what are the risk factors for death from Influenza A(H1N1) in Brazil during the epidemic?” we decided to use models that encompass the various dimensions studied, biological characteristics, pre-existing diseases, and hospital assistance received by patients. The first model included only the first two dimensions, the second model also included service transfer, and the third model also considered ICU admissions.

In Model 1 (Table 4), it is evident that biological factors such as age and obesity can influence the risk of dying from influenza A(H1N1), but it is also influenced by pre-existing diseases such as congestive heart failure and diabetes mellitus. The results of Model 2 indicate that assistance can also independently influence the risk of the studied outcome. Finally, in the model that includes ICU admission, many factors lose their ability to discriminate the risk of death, which does not mean that these are not risk factors, but that the risks of these biological and clinical conditions are probably represented by the severity of the critical condition requiring intensive care.

**Table 4:** Biological, clinical, and care risk factors for death due to influenza A(H1N1).

Risk factor	Model 1		Model 2		Model 3	
		
	OR_adjusted_	95%CI	OR_adjusted_	CI 95%	OR_adjusted_	95%CI
Biological characteristics						
Age (years)	1.015	0.998–1.031	1.017	1.000–1.035	--	--
Are you obese?	2.631	0.939–7.367	2.296	0.785–6.708	--	--
Pre-existing diseases						
Congestive heart failure	10.326	1.012–105.347	6.851	0.659–71.189	--	--
Diabetes mellitus	8.508	0.890–81.333	7.122	0.694–73.001	24.216	1.772–330.811
Respiratory tract diseases	2.182	1.022–4.657	2.042	0.929–4.490	--	--
Hospital care						
Has it been transferred from the service?			3.014	1.620–5.609	1.897	0.891–4.038
Were you admitted to the ICU?					29.468	13.137–66.098

ICU: intensive care unit.

## DISCUSSION

Our results indicate that biological factors, such as age and obesity, are related to the risk of dying from influenza A(H1N1). There is also evidence that pre-existing diseases, such as congestive heart failure, respiratory disease, and diabetes mellitus, are associated with death from this cause. However, our study reveals that the transfer of hospitalization service and ICU stay are conditions that imply that the worsening of the clinical condition increases the risk of death due to influenza A(H1N1).

The set of signs presented by the participants are typical of respiratory syndrome, and the absence of significant difference in the set of signs and symptoms indicates the adequacy of the study selection process regarding the eligibility criteria. This profile was similar to that observed in other investigations conducted in Brazil^9,10^.

Ribeiro et al.^10^ conducted a case–control study with 193 reported and confirmed deaths of patients with influenza A(H1N1), and 386 patients hospitalized during the study period with reported and confirmed influenza A(H1N1) who recovered. The distribution of cases and controls according to demographic variables indicated that the median age of cases was higher than that of controls, and the age group from 18 to 59 years had a higher risk compared with those aged less than 18 years. The elderly also had a significant risk of dying from influenza A(H1N1).

During the 2009 epidemic, another case–control study was conducted in Brazil. Yokota et al.^9^ carried out an investigation in 11 hospitals in four cities (Passo Fundo, Caxias do Sul, Santa Maria, and Uruguaiana) in Rio Grande do Sul, Brazil. The study included 157 patients, 52 who died and 105 who survived. A total of 136 patients sought treatment before hospitalization, and obesity was the most common underlying medical condition (38%). Among the obese patients, 19 (36%) had other risk factors for influenza complications. Diabetes was the most frequent medical condition (21%) in obese individuals.

In our investigation, most of the factors studied were not associated with the outcome of death from influenza A(H1N1), so the results of the statistical analysis of the data included only the factors whose underlying theory or exploratory analysis indicated a possible association. Although obesity was not included in the final model, it was associated with death from influenza A(H1N1) in our study.

Sun et al.^11^ performed a systematic review combining results from 22 articles involving 25,189 laboratory-confirmed patients. Pooled estimates indicated that obesity significantly increased the risk of death from influenza A (H1N1). However, the authors indicated that they found a significant interaction between early antiviral treatment and obesity. After adjusting for early antiviral treatment, the relationship between obesity and poor outcomes disappeared. This situation may also have occurred in our study. A review study carried out by Karlsson et al.^12^ showed that obesity is associated with decreased lung function, as well as the development of chronic respiratory conditions, and risk factors for the development of severe influenza-associated infection.

Balaganesakumar et al.^13^ conducted a case-control study in Tamil Nadu, India, with 280 participants (70 patients who died and 210 patients who recovered) between July and December 2010. In this study, fever (96%) and cough (79%) were the most common symptoms, whereas 12% of patients had dyspnea in 85% of deaths. This profile was similar to that of our study, with a higher proportion of fever (94.1%) and dyspnea (80.3%) than the other signs. In the Indian study^13^, the chances of death were higher in diabetic and obese patients who received treatment from private services and were treated with corticosteroids.

Martinez et al.^14^ conducted an observational study with patients aged ≥ 18 years from 12 Catalan hospitals from 2010 to 2016. A total of 1,726 hospitalized patients were included, 595 (34.5%) were admitted to the ICU, and 224 (13.0%) died. Age groups 65 to 74 and ≥75 years were associated with an increased risk of death for all types and subtypes, especially for influenza B. Immunodeficiency was associated with death for influenza B and subtype A(H1N1). These findings are similar to our study, but immunodeficiency was not observed in the model.

Mata-Marin et al.^15^ conducted a case–control study to determine the risk factors associated with death from influenza A(H1N1), as detected by RT-PCR. The study included patients over the age of 18 years who were treated at the Hospital de Infectologia, Centro Médico Nacional “La Raza”, in Mexico City, between April and November 2009. Cases were patients who died during hospitalization, whereas controls were discharged from the hospital. A total of 33 patients met the eligibility criteria. Risk factors associated with mortality were male sex, delayed medical care >3 days, delayed influenza therapy >3 days, intensive care unit (ICU) admission, and creatinine levels >1.0 mg/dL when admitted to the hospital. After adjusting for a logistic regression model, delay in medical care and ICU stay were the only predictors of mortality.

Sanya-Olalla Peralta et al.^16^ studied factors associated with the risk of death in cases of 2009 (H1N1) pandemic for patients hospitalized in the ICU in Spain. Of the 1,231 cases admitted to the ICU, 271 died. The median age was 40 years, and 76.3% of patients had some previous disease, with respiratory disease being the most frequent (34.1%), followed by morbid obesity (18.8%). In multivariate analysis, cancer, immunodeficiencies, and morbid obesity were significantly associated with death in adults.

Another Brazilian study^17^ addressed severe maternal morbidity due to respiratory diseases and the impact of the 2009 influenza A(H1N1) pandemic in Brazil. The objective of this study was to evaluate the burden of respiratory diseases, considering the time of the pandemic within the scope of the Brazilian Network for the Surveillance of Severe Maternal Morbidity, and the factors associated with the worst maternal outcome. Women with severe complications from respiratory disease identified as suspected or confirmed cases of influenza A(H1N1) or respiratory failure were compared with those with other causes of severe morbidity. A review of suspected influenza A(H1N1) cases classified women as untested, tested positive, and tested negative, comparing their results.

Ribeiro et al.^18^ also studied the factors associated with death and described the gestational outcomes in pregnant women with A(H1N1)pdm09 influenza. severe acute respiratory illness (SARI) in the State of São Paulo from June 9 to December 1, 2009. They investigated 48 cases and 185 controls. The results of this study indicate that early treatment can prevent unfavorable outcomes for pregnant women and their children and reinforce the need for adequate training of doctors for the clinical management of pregnant women and early administration of antiviral treatment. Other Brazilian studies have also indicated similar results^19,20^.

No other Brazilian study was found in the scientific literature that had a scope and methodological approach similar to that used in our investigation. The challenge of identifying risk factors for death from H1N1 influenza includes the need to consider the medical and hospital care provided to patients.

If, on the one hand, it is worth stating that adequate and timely assistance can prevent deaths, it must also be considered that the most critical patients are those who receive intensive care. Therefore, we questioned whether the transfer of patients to more specialized services and the admission to an intensive care unit were associated with the risk of dying from H1N1 influenza. The occurrence of “reverse causality” is plausible if these relationships are considered causal. This study intended to identify risk markers for death from this disease, although we use the term risk factor to indicate these studied characteristics. As expected, i.e., hypothesized, both transferring services and hospitalization were strongly associated with death from influenza. The strength of the association found for ICU admission was the highest found in our investigation.

Regarding the ability of this study to assess the influence of the participants’ vaccination status on the risk of dying from A(H1N1) influenza, it is worth considering that the low proportion of vaccinated individuals in the sample may have had a negative influence. Despite the result indicating protection when calculating the measure of effect adjusted for the other factors, only 5% of the participants were vaccinated, and as already mentioned, the expected effect of the immunization campaign may not yet have been detected. In our study, participants vaccinated for influenza were not more among the cases (deaths) than non-vaccinated ones. In fact, in March 2010, the federal government started a vaccination campaign. In just three months, using the vaccines purchased and new batches manufactured by the Butantan Institute, Brazil managed to vaccinate 92 million people. The federal government expected to vaccinate 80% of the priority groups but managed to reach 88%. The success is due to the mobilization effort and the strategy of implementing vaccination points in schools, public offices, workplaces, and even public roads^21^.

Mahmud et al.^22^ performed a population-based case–control study to evaluate the effectiveness of pandemic vaccines against influenza A(H1N1) during the 2009 mass vaccination campaign in Manitoba (Canada). Cases were individuals who tested positive for influenza A by PCR (n = 1,435). Controls were individuals with negative results for influenza A and B (n = 2,309). Results indicated that the adjuvanted H1N1 vaccine was 86% effective (95%CI 75–93%) in preventing H1N1 infections when vaccination occurred ≥ 14 days before testing. Although it was not possible to study the effectiveness of the vaccine in our study, there seems to be no doubt about its benefits. Lansbury et al.^23^ conducted a meta-analysis on the effectiveness of 2009 pandemic influenza A(H1N1) vaccines and concluded that both adjuvanted and unadjuvanted monovalent vaccines were effective in preventing influenza. Overall, the vaccines were also effective against influenza-related hospitalization. For both outcomes, adjuvanted vaccines were more effective in children than in adults.

This study had limitations such as underreporting of SARS, but as the epidemic progressed, it may have decreased. The quality of hospital record data may have also influenced the results. The strategy used to minimize the quality of data extraction was the careful review of this process by the supervisor.

Our study had to consider the need to address aspects of various dimensions to identify the risk factors for death from influenza A(H1N1) and the three final models revealed the importance of the groups of factors studied. These approaches complement each other in a way because in the absence of a robust theoretical model to study this question, highlighting the importance of these characteristics is only possible with this analytical approach.

The risk factors identified in this investigation not only allowed for subsidizing the elaboration of clinical conducts but also indicated important aspects for facing new influenza epidemics that are likely to occur in our country.
